# Prophylactic lithium treatment and cognitive performance in patients with a long history of bipolar illness: no simple answers in complex disease-treatment interplay

**DOI:** 10.1186/s40345-014-0016-7

**Published:** 2014-12-24

**Authors:** Andrea Pfennig, Martin Alda, Trevor Young, Glenda MacQueen, Janusz Rybakowski, Aleksandra Suwalska, Christian Simhandl, Barbara König, Tomas Hajek, Claire O’Donovan, Dirk Wittekind, Susanne von Quillfeldt, Jana Ploch, Cathrin Sauer, Michael Bauer

**Affiliations:** Department of Psychiatry and Psychotherapy, Carl Gustav Carus University Hospital, Technische Universität Dresden, Fetscherstrasse 74, 01307 Dresden, Germany; Department of Psychiatry, Dalhousie University, Halifax, Canada; Department of Psychiatry, University of British Columbia Faculty of Medicine, Vancouver, Canada; Department of Psychiatry, Faculty of Medicine, University of Calgary, Calgary, Canada; Department of Adult Psychiatry, Poznan University of Medical Sciences, Poznan, Poland; Department of Psychiatry, Hospital Neunkirchen, CS now: Bipolar Centre, Vienna, Austria; Department of Psychiatry and Psychotherapy, Charité Berlin, Campus Mitte, Berlin, Germany; International Group for the Study of Lithium-Treated Patients (IGSLi), Berlin, Germany

**Keywords:** Lithium, Bipolar disorder, Cognition

## Abstract

Cognitive impairment in patients with bipolar disorder (BD) is not restricted to symptomatic phases. It is also present in euthymia. There is evidence of differences in the brain’s structure between bipolar patients and healthy individuals, as well as changes over time in patients. Lithium constitutes the gold standard in long-term prophylactic treatment. Appropriate therapy that prevents new episodes improves the disease’s course and reduces the frequency of harmful outcomes. Interestingly, preclinical data suggest that lithium has a (additional) neuroprotective effect. There is limited data on its related effects in humans and even less on its long-term application. In this multi-center cross-sectional study from the International Group for the Study of Lithium-treated Patients (IGSLi), we compared three groups: bipolar patients without long-term lithium treatment (non-Li group; <3 months cumulative lithium exposure, ≥24 months ago), bipolar patients with long-term lithium treatment (Li group, ongoing treatment ≥24 months), and healthy subjects (controls). Strict inclusion and exclusion criteria were defined; the inclusion criteria for patients were diagnosis of BD types I or II, duration of illness ≥10 years, ≥5 episodes in patient’s history and a euthymic mood state. Neurocognitive functioning was assessed using the Wechsler Adult Intelligence Scale-Revised (WAIS-R), the California Verbal Learning Test (CVLT), and a visual backward masking (VBM) task. A total of 142 subjects were included, 31 in the non-Li and 58 in the Li group, as well as 53 healthy controls. Treated patients with long-standing BD and controls did not differ significantly in overall cognitive functioning and verbal learning, recall, and recognition; regardless of whether lithium had been part of the treatment. Patients, however, demonstrated poorer early visual information processing than healthy controls, with the lithium-treated patients performing worse than those without. Our data suggest that bipolar patients with a long illness history and effective prophylactic treatment do not reveal significantly impaired general cognitive functioning or verbal learning and memory. However, they are worse at processing early visual information. Accompanying volumetric and spectroscopic data suggest cell loss in patients not treated with lithium that may be counterbalanced by long-term lithium treatment.

## Background

In patients with bipolar disorders, treatment strategies aim at improving the course of this often life-long and devastating disease. The quality of cognitive functioning is considered one of the key variables predicting health-related quality of life, apart from the illness’s severity and clinical course and the presence of residual affective symptomatology (see review Andreou and Bozikas 2013). It is known that cognitive impairment is not restricted to symptomatic phases, but that it also accompanies euthymia in a substantial number of bipolar patients, even though the published evidence is heterogeneous with regard to the specific functions affected, degree of impairment, and the degree of variability explained by the disorder itself compared to that attributed to moderating and mediating variables (Jamrozinski 2010). There seems to be a correlation between the duration of illness (DOI) as well as the number of illness episodes (especially depressive ones) and the degree of impairment (Bearden et al. 2001, Bora et al. 2010). Data on the latter, however, are discordant and even the causal direction is not yet clear (Martino et al. 2014).

Another scale of cognitive impairment is seen in the development of dementia. Interestingly, the risk for mild cognitive impairment (MCI) and dementia seems to be higher in bipolar patients (the same is true for MDD patients) (Reischies and Neu 2000; Jorm 2001; Kessing and Nilsson 2003) and it continues to rise with the number of the disease’s past episodes (Kessing and Andersen 2004). Regarding the basis of cognitive functioning, evidence has been forthcoming in recent years of differences in brain structure between bipolar patients and healthy individuals, as well as changes over time in patients. A meta-analysis of 16 voxel-based morphometry (VBM) studies found neuroanatomical abnormalities in the right prefrontal cortex, anterior temporal cortex, insula, and claustrum (Selvaraj et al. 2012).

Pharmacological treatment with lithium has been proven to possess antimanic, prophylactic, and antisuicidal properties. Lithium constitutes the gold standard in long-term prophylactic treatment. Appropriate therapy that prevents new episodes does in itself improve the disease’s course and reduces harmful outcomes including substantial neurocognitive impairment. Interestingly, preclinical (cell culture and animal) data suggest a (additional) neuroprotective effect mainly by preventing apoptosis and increasing the excretion of neurotrophin (Bauer et al. 2003). There is limited data on the effects of lithium treatment on these functions in humans. Short-term exposure to lithium increased gray-matter volume throughout the brain in bipolar patients (Moore et al. 2000). Almost no data exist on long-term exposure specifically assessing the influence of the duration of lithium treatment. The International Group for the Study of Lithium-treated Patients (IGSLi) therefore conducted a cross-sectional study with strict inclusion and exclusion criteria to clarify whether there is a reason to suggest that lithium can prevent neurocognitive impairment and/or structural and functional changes in specific cortical areas in order to decide whether a prospective study should be planned. Since the hippocampus plays a substantial role in regulating adult neurogenesis (Eriksson et al. 1998; Kempermann and Gage 1999), and cell loss in this area greatly impairs neuronal plasticity, the structure and function of this brain area were of main interest. Whether the proposed preventive effects of lithium are exerted on the aforementioned structures to prevent changes and thus cognitive impairment (and/or dementia) is not clear so far. On the contrary, some studies did reveal cognitive side effects in lithium-treated patients (Goldberg and Chengappa 2009; Pachet and Wisniewski 2003; Wingo et al. 2009).

In the present paper we describe the cognitive performance results of the IGSLi study and discuss potential structure-function interactions using imaging data (Hajek et al. 2012, Hajek et al. 2014).

## Methods

### Design

In this cross-sectional study integrating lifetime information, we compared three groups of individuals: bipolar patients without long-term lithium treatment (non-Li group), bipolar patients with long-term lithium treatment (Li group), and neuropsychiatrically healthy subjects (controls). Long-term lithium treatment was defined as current ongoing treatment for at least 24 months. Patients having experienced no or fewer than three months of cumulative lithium exposure at least 24 months ago were included in the group without long-term lithium treatment.

### Subjects

Patients were recruited via outpatient clinics at the five cooperating university hospitals and their cooperating local psychiatrists. Healthy controls were recruited via notices. The study centers where Berlin and Dresden in Germany, Halifax in Canada, Poznan in Poland, and Neunkirchen in Austria.

Inclusion criteria for patients included diagnosis of BD types I or II (verified with the SCID), a DOI of at least 10 years, and the occurrence of at least five episodes in the disease history (including (hypo-)manic, depressive, and/or mixed episodes). Patients had to be in a euthymic mood state (HAM-D-17 < 7, YMRS <5, CGI-BP ≤3) and had to have been free of significant symptoms for at least 4 months. They had to be mentally and physically sound and have adequate language skills to participate in the study. They also had to return a written informed consent form.

Exclusion criteria for bipolar patients included a history of more than one lifetime course of ECT (12 or more bilateral treatments), ECT in the last 12 months, another comorbid psychiatric disorder with the following exceptions: comorbid anxiety (but not posttraumatic stress disorder (PTSD)) and/or personality disorder (but not borderline personality disorder) were allowed, as long as BD was the primary diagnosis. History of substance abuse was allowed as long as there was no active abuse in the last 12 months. Current psychotic features or acute suicidality were further exclusion criteria.

Patients in the Li group could be treated with only one additional psychotropic drug (but not with first-generation antipsychotics, clozapine, or tricyclic antidepressant). Patients in the non-Li group were allowed to take two psychotropic drugs not in the aforementioned classes. Current treatment with benzodiazepines at doses exceeding 2 mg/d equivalent of lorazepam or clonazepam or higher was not allowed. Patients had to have been free of significant changes in their medication for at least the previous 3 months.

Furthermore, patients were excluded if they suffered from a medical illness that was not corrected or stable (e.g. hypothyroidism, vitamin B12 insufficiency, diabetes mellitus, and hypertension had to be in stable condition) or was very severe (e.g. carcinoma, metastases, and all neurological illnesses including dementia). Patients with metal implants or metallic artifacts were not included in the imaging part of the study.

Neuropsychiatrically healthy individuals were eligible provided they were in sound mental and physical condition and possessed adequate fluency in the participating center’s language to participate in the study. The control group’s exclusion criteria included a personal history of any psychiatric illness (verified with the SCID, history of substance abuse was allowed as long as there was no active abuse in last 12 months). They could not have a family history in first-degree relatives involving the DSM IV diagnoses of BD, unipolar recurrent depressive disorder, schizophrenia or schizoaffective disorder, or a suicide of a first-degree relative. As in bipolar patients, unstable or very severe medical illnesses, current intake of 2 mg/d equivalent of lorazepam or clonazepam or higher, significant changes in medication within the last 3 months and for the imaging part, and any metal implants and metallic artifacts were not allowed.

The three groups of subjects were matched for age as closely as possible.

### Study procedure

Study assessments were conducted on two to three consecutive days, whenever possible. First of all, sociodemographic data were collected, the diagnosis (SCID) and illness history were established (including the use of the LCM), euthymia was confirmed (HAM-D-17, Hamilton 1960; YMRS, Young et al. 1978; CGI-BP, Spearing et al. 1997), a cursory physical examination was conducted, and routine laboratory parameters were monitored including thyroid hormone, vitamin B12, and folate levels as well as lithium serum levels in the long-term lithium patients. Health-related quality of life was assessed using the Medical Outcome Studies Short-Form General Health Survey (SF-36, Ware et al. 1993, Ware et al. 1994), the Quality of Well-Being Scale, Self-rating version (QWB-SA, Kaplan et al. 1998), and data from the BDI (Beck et al. 1961).

### Neuropsychological assessment

Neurocognitive functioning was assessed in tasks where impaired performance in patients with BD was evident and that seemed to involve brain areas crucial to the study questions (namely, the Wechsler Adult Intelligence Scale-Revised (WAIS-R; in German HAWIE, Tewes 1991), the California Verbal Learning Test (CVLT, Delis et al. 1988), and a visual backward masking (VBM, MacQueen et al. 2004) task).

Briefly, the WAIS-R is the primary clinical instrument to measure overall neurocognitive function. It consists of six verbal and five performance subtests. The verbal subtests assess information (29 questions of general knowledge), comprehension (16 questions which focus on issues of social awareness), arithmetic (14 mental arithmetic brief story-type problems), digit span (sets of digits to repeat initially forwards then backwards), similarities (subjects asked to say how two seemingly dissimilar items might in fact be similar), and vocabulary (subject is asked to define 35 words). The performance part consists of tests arranging pictures (the subject is asked to arrange ten sets of small pictures into a logical sequence), picture completion (20 small pictures that all have one vital detail missing), block design (involves putting sets of blocks together to match patterns on cards), object assembly (four small jig-saw type puzzles), and digit symbol (Involves copying a coding pattern).

The CVLT is an established test of verbal learning and of recall (encoding and retrieval) and recognition of the learned content. A list of 16 words (belonging to four semantic categories) is read five times with memory being assessed after each trial. Immediately after the fifth trial, the subject is read an interference list and asked to recall it. A short delayed recall test is conducted immediately after recalling the interference list, where the participant is asked to recall the words in the list read initially. Cues are then provided for the four semantic categories to facilitate recall. A long delayed recall test is presented after a 20-min interval where the subject works with non-verbal tasks (in our study, the VBM). Finally, a ‘yes-no’ recognition test consisting of the 16 items on the initial list, eight from the interference list, and 20 random distractor items is presented. This test is sensitive to temporo-hippocampal dysfunction (Turner et al. 2012, Chepenik et al. 2012).

The VBM task assesses early visual information processing in interpreting the interaction of transient (movement and location) and sustained (more detailed information for recognition) visual pathways. In the computerized VBM task, a briefly presented target (first stimulus) is covered by an uninformative mask (second stimulus) with inter-stimulus intervals (ISI) of 14, 29, 43, 57, 86, and 114 ms. The subject is asked to point with a joystick in the direction in which the target was presented (up, down, left, right). Performance on this task is mediated by magnocellular visual pathways projecting to the dorsal occipito-parietal and frontal regions.

These neurocognitive tests were conducted beginning with the WAIS-R, followed by the CVLT with the VBM in the 20-minute break required. Patients had to postpone medication intake on the test day until the end of testing.

### Study endpoints

Primary endpoints of this study were the neuropsychological functioning scores in the neuropsychological tests (focusing on CVLT) and the volume of the hippocampus in MRI (Hajek et al. 2013). Secondary endpoints included N-acetylaspartate (NAA) levels (plus Cho and Cr) in prefrontal cortex areas (Hajek et al. 2012) and health-related quality of life.

### Statistical analysis

The sample size estimation was conducted based on the following assumptions: For euthymic bipolar patients and healthy subjects, we reviewed published data on CVLT performance. Martinez-Aran et al. (2004) showed that euthymic patients functioned about 17% worse than controls (trials 1 to 5 mean remembered words 45.1 (SD 11.4) and 54.4 (SD 9.6)). Other CVLT data (e.g. Fleck et al. 2003) and those from other neurocognitive tasks do support the assumption of a poorer performance by at least 10%. Unpublished pilot data from the IGSLi group collected before planning this study indicated neurocognitive functioning in long-term lithium-treated bipolar patients that did not differ from that of healthy controls. With an alpha of 0.05 and a β of 0.2, 27 subjects in each of the three groups to be studied should suffice to enable the detection of significant differences (nQuery Advisor © 4.0). Since our second primary endpoint was hippocampal volume, we conducted a separate sample size estimation. Based on published data from Blumberg et al. (2003), bipolar patients demonstrated a reduction in hippocampal volume by about 5% compared to healthy controls (3,039 (SE 68) and 3,209 (SE 54) mm^3^). The whole gray matter’s volume in lithium-treated bipolar patients was higher than that in healthy controls and untreated bipolar patients (747.9 cm^3^, SD 69.8, 675.8 cm^3^, SD 61.8 and 639.2 cm^3^, SD 91.2; Blumberg et al. 2003). Using these data and assuming the same mean of 3,209 mm^3^ for lithium-treated patients as for healthy controls and an SE of 60, an alpha of 0.05 and a β of 0.2 and at least 70 subjects in each of the three groups under investigation should enable us to detect significant differences (nQuery Advisor © 4.0).

In the statistical analysis of the project part presented here, we made a three-group comparison for each cognitive test. For the CVLT and WAIS-R, separate multivariate analyses of covariance (MANCOVA) with age, sex, education, duration of illness, and center as covariates were used. Dependent variables were predefined as being trial 1 (words remembered after once reading the list, mainly evaluates auditory attention span), trials 1 to 5 (summary score of words remembered after reading the list five times, global measure of verbal learning, and immediate free recall), recognition hits (helps differentiating impairment in encoding and recall, with impairment in the latter when free recall is impaired, but not recognition), false positives (response bias, liability to answer ‘yes’), and intrusions (measure of memory, associated with hippocampal function) from the CVLT (MANCOVA 1) as well as verbal part, non-verbal part, and total score from the WAIS-R (MANCOVA 2). For the VBM data separate MANCOVA with repeated measurements with reaction time (MANCOVA 3) and error rate (MANCOVA 4) in the six ISI (14, 29, 43, 57, 86, and 114 ms) as dependent variables and age, sex, education, duration of illness, and center as covariates were applied. Separate group comparisons were made for each individual interval time using *t*-tests. Because of multiple testing, only *p* < 0.001 is considered significant. The potential influence of duration of illness on the cognitive performance was informatively studied using correlation analysis. In case of substantial correlations, we applied regression analysis.

All study subjects were extensively informed about the study and signed an informed consent form. The study was approved by the local ethics committees at all participating study centers.

## Results

We enrolled a total of 142 subjects in the study: 31 bipolar patients in the non-Li group, 58 bipolar patients in the Li group, and 53 healthy controls. The recruitment figures from each study center are listed in Table 1. Overall, groups were comparable regarding sociodemographic variables (Table 2). Matching for age was not entirely successful. Since differences in age, sex, and education have been suggested to influence the data substantially, we adjusted the statistical analysis to accommodate those variables (apart from the center variable).

**Table 1:** **Recruitment figures of the individual study centers**

	**Non-Li group**	**Li group**	**Controls**	**Total**
Berlin	6	18	20	44
Dresden	5	5	2	12
Poznan	5	10	10	25
Halifax	8	15	11	34
Neunkirchen	7	10	10	27
Total	31	58	53	142

**Table 2:** **Sociodemographic variables of the sample**

	**Non-Li group**	**Li group**	**Controls**
n	31	58	53
Age in years, mean (SD)	45.3 (12.9)	49.4 (11.3)	44.8 (8.9)
Men, n (%)	32%	38%	40%
Family status, n (%)			
Single	22%	26%	27%
Relationship	67%	68%	73%
Educational level, n (%)			
University degree	29%	35%	39%
College/Abitur	46%	49%	39%
High school	25%	12%	21%
No degree	0%	4%	2%
Employment status, n (%)			
Employed	58%	48%	73%
Early pensioner	15%	19%	7%

The patients included in the study already had a long history of the disease with at least five episodes of the illness. See Table 3 for clinical characteristics.

**Table 3:** **Clinical characteristics of the patient groups**

	**Non-Li group**	**Li group**
Duration of illness in years, mean (SD)	20.1 (10.1)	23.8 (9.0)
Bipolar I, %	56%	59%
Medical comorbidity, %		
High blood pressure and any cardiovascular disease	28%	16%
Diabetes mellitus	4%	7%
Thyroid disorder	8%	36%
Asthma and any lung disease	12%	5%
Rheumatism, arthritis	16%	5%
Medication, %		
Lithium	0%	100%
Valproate	35%	5%
Carbamazepine	17%	7%
Lamotrigine	21%	7%
Atypical antipsychotics	41%	12%
Non-tricyclic AD	45%	21%
Benzodiazepines	10%	2%

AD, antidepressant.

As our inclusion and exclusion criteria make clear, patients were allowed to take one psychotropic drug in addition to lithium or two psychotropic drugs (group without long-term lithium treatment). As illustrated in Table 3, patients in the non-Li group took all permissible drug classes at a higher frequency than the Li group patients: Valproate (35%), atypical antipsychotics (41%), and non-tricyclic antidepressants (45%) were the drug classes that the non-Li group used most frequently. The Li group patients had taken lithium for an average of 11.1 ± 8.2 years. Lithium levels on the days of neurocognitive testing were within the therapeutic range (0.66 ± 0.17 mmol/l).

**Figure 1:**
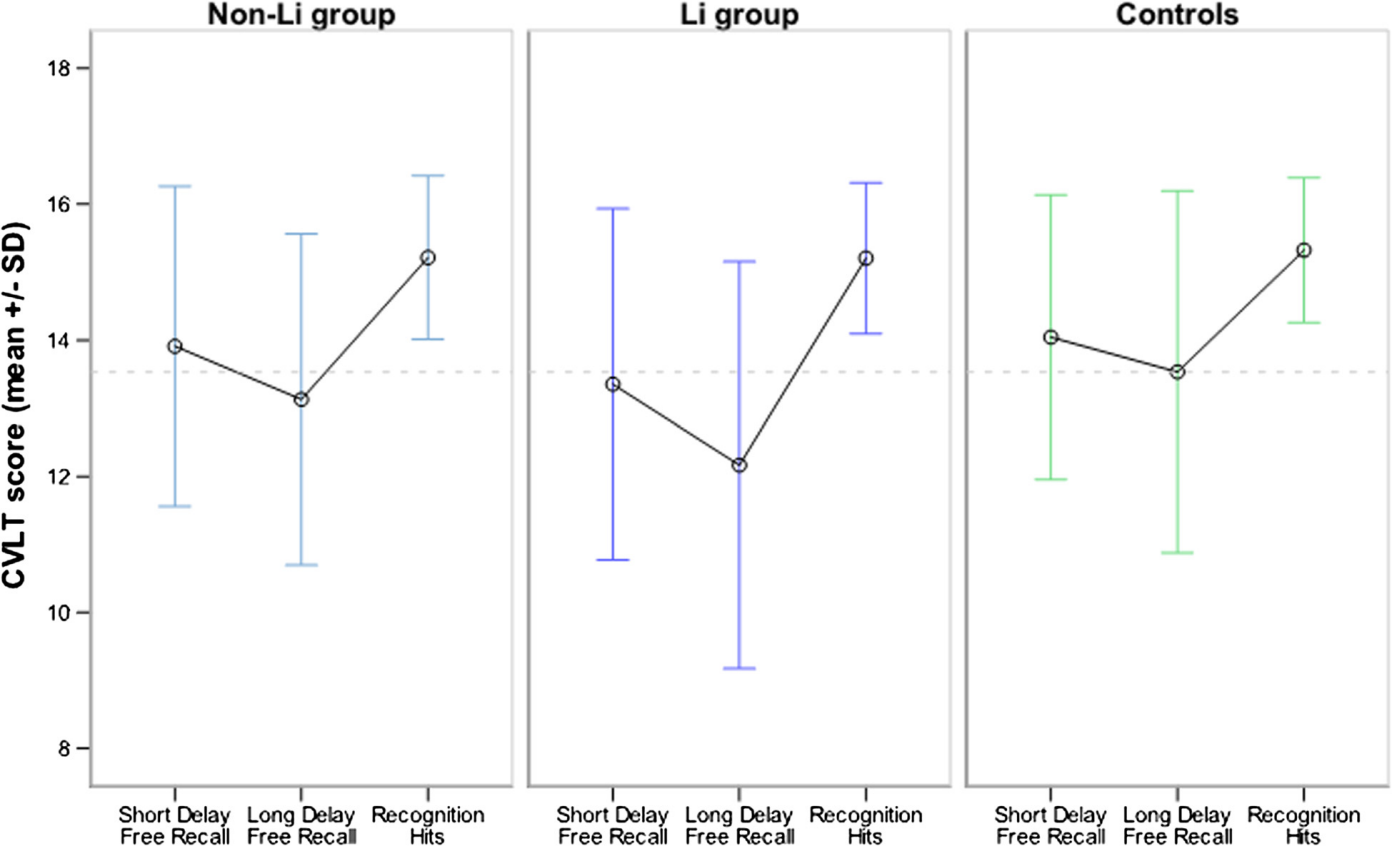
**Short delay free recall, long-delay free recall, and recognition hits in the three groups.** Dotted line: comparison to mean of long-delay free recall score in healthy controls.

We identified no significant differences after comparing the verbal learning and memory (CVLT) results from the three groups (Table 4 and Figure 1). Excluding patients with a university degree from the analysis (to analyze whether differences between the groups were diminished because many patients performed on a very high baseline level) did not alter the results (data not shown).

**Table 4:** **Corrected results of the individual patient groups and controls in the CVLT**

**Variables**	**Non-Li group**	**Li group**	**Controls**	**F**	***p***
**(Mean ± SE)**	**(n = 23)**	**(n = 45)**	**(n = 34)**		
Trial 1	7.53 (0.50)	8.19 (0.44)	8.31 (0.66)	0.825	0.442
Trials 1 to 5	59.39 (2.22)	58.06 (1.94)	59.23 (2.93)	0.151	0.860
Recognition hits	15.28 (0.27)	15.25 (0.24)	15.21 (0.36)	0.006	0.994
False positives	0.49 (0.38)	1.30 (0.33)	1.04 (0.50)	1.889	0.157
Intrusions	3.28 (0.82)	4.15 (0.71)	2.88 (1.08)	0.602	0.550

**Figure 2:**
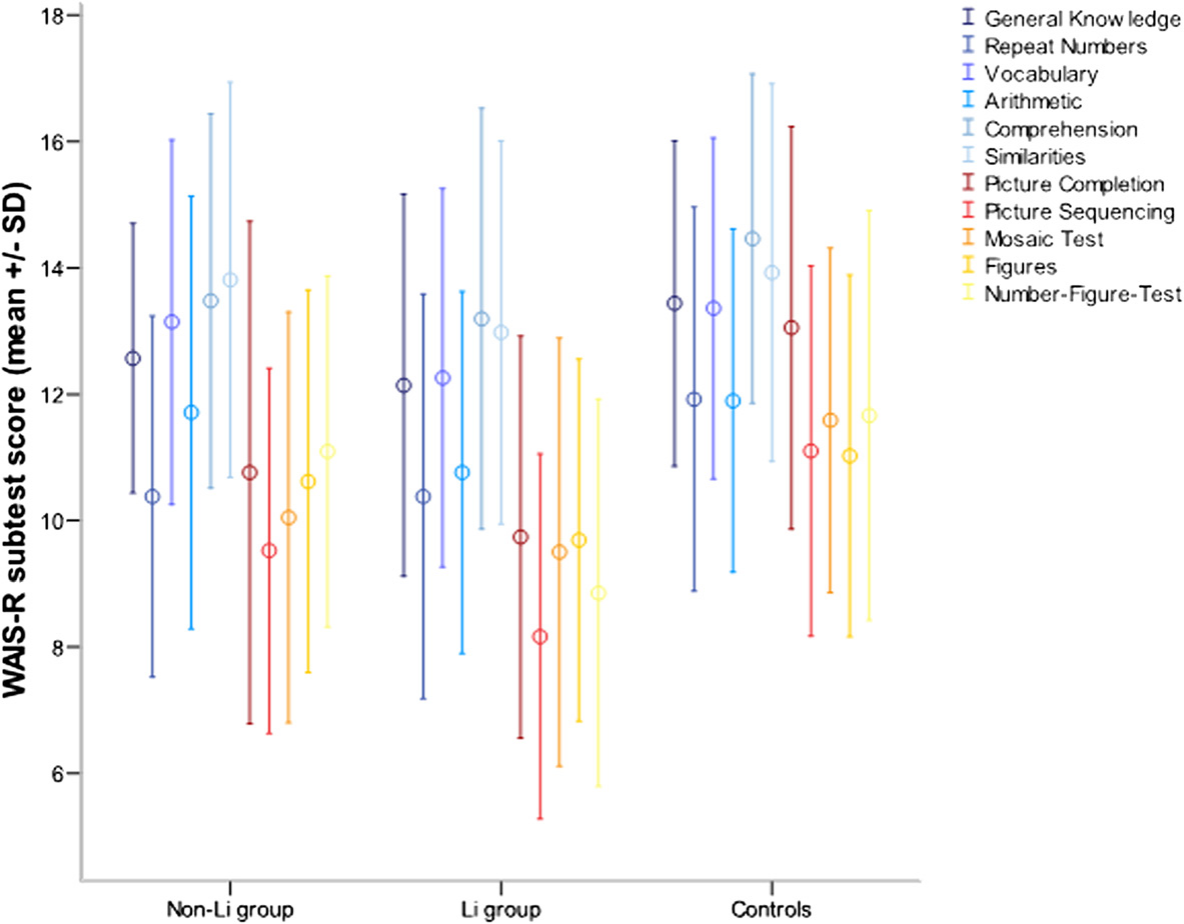
**WAIS-R subtest scores.** WAIS-R subtest scores of patients without and with long-term lithium treatment compared to healthy controls adjusted for age, sex, education, duration of illness, and center dark to light blue: subtests of the verbal part, red to yellow: subtests of the performance part.

Regarding overall cognitive functioning, the three-group comparison yielded no significant difference in results from the verbal and performance parts or from the WAIS-R summary score (Table 5). As with the CVLT data, the results were not altered by excluding patients with a university degree from the analysis (data not shown). When examining differences in individual tests within the verbal and performance parts, we noticed that no subtest stood out of the pattern of impairment (Figure 2).

**Table 5:** Corrected results of the individual patient groups and controls in the WAIS-R

**Variables**	**Non-Li group**	**Li group**	**Controls**	**F**	***p***
**(Mean ± SE)**	**(n = 20)**	**(n = 40)**	**(n = 34)**		
Verbal part	77.88 (2.86)	73.75 (2.79)	73.12 (3.57)	0.946	0.393
Performance part	53.36 (2.41)	51.22 (2.35)	52.11 (3.00)	0.269	0.765
Summary score	131.16 (4.38)	125.10 (4.27)	126.00 (5.46)	0.732	0.484

**Figure 3:**
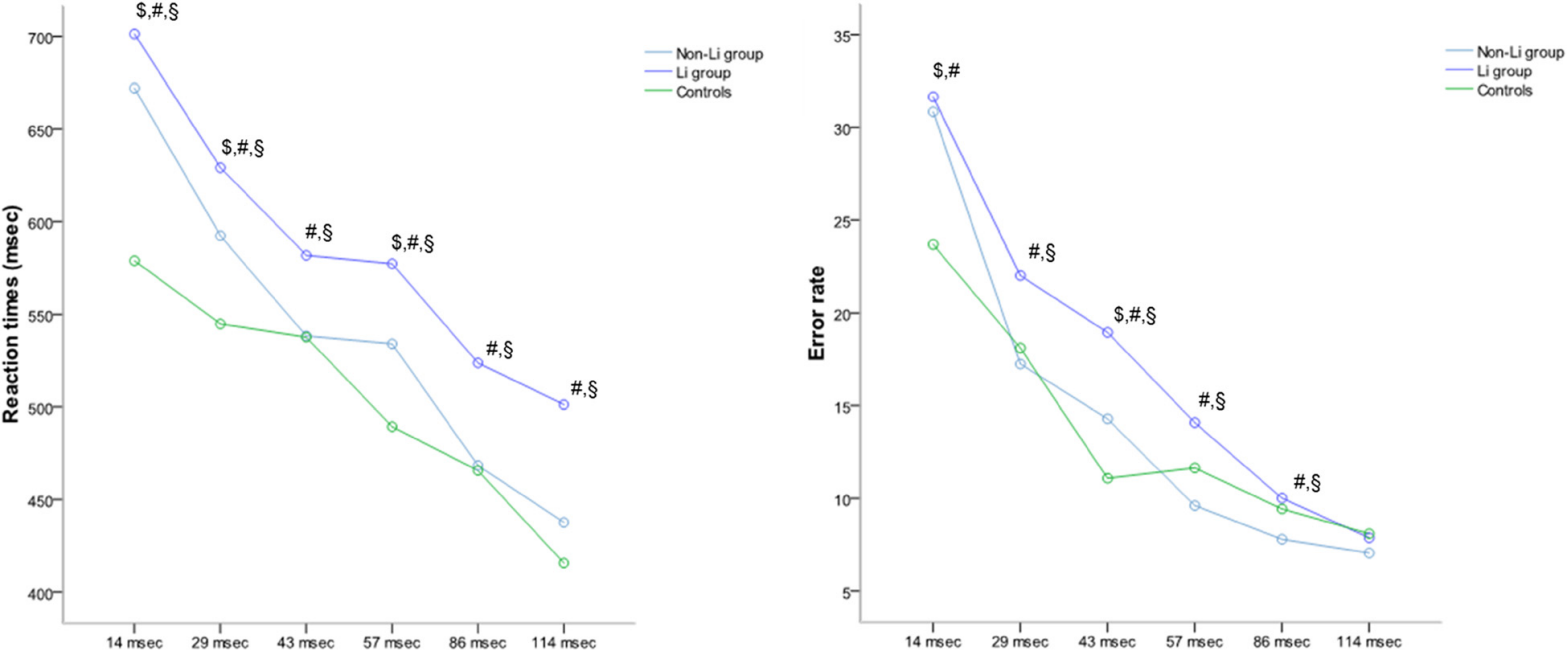
**Reaction times and error rates.** Reaction times (left graph) and error rates (right graph) of patients without and with long-term lithium treatment and healthy controls adjusted for age, sex, education, duration of illness, and center, *p* < 0.001: ^$^non-Li group vs. healthy controls, ^#^Li group vs. healthy controls, ^§^Li group vs. non-Li group.

In the visual backward masking task assessing early visual information processing, as expected, all subjects responded quicker and had lower error rates the longer the interval between the target’s presentation and covering by the mask was (ISI). We observed no statistically significant difference among the three groups in the overall performance development in terms of reaction times or error rates (F = 1.062 and *p* = 0.349; F = 0.470 and *p* = 0.626). All the ISI patients on lithium treatment were significantly slower than the non-lithium patients and healthy controls. At 14 ms, they made significantly more errors than healthy controls; at 29, 43, 57, and 86 ms, they made still more errors than the non-lithium patients. Non-lithium patients were significantly slower and made more errors than healthy controls at 14 and 57 ms; at 29 ms, they were slower; and at 43, they made more errors (Table 6 and Figure 3).

**Table 6:** Corrected results of the individual patient groups and controls in the VBM

**Variables**	**Non-Li group**	**Li group**	**Controls**	***p***	***p***	***p***
**(Mean ± SE)**	**(n = 27)**	**(n = 50)**	**(n = 43)**	**Non-Li vs. controls**	**Li vs. controls**	**Non-Li vs. Li**
*Reaction times*						
RT 14 ms	664.82 (40.33)	698.92 (36.34)	584.08 (49.71)	<0.001	<0.001	<0.001
RT 29 ms	588.55 (40.08)	627.52 (36.12)	546.59 (49.40)	<0.001	<0.001	<0.001
RT 43 ms	535.46 (35.88)	581.81 (32.33)	541.46 (44.23)	0.433	<0.001	<0.001
RT 57 ms	530.46 (33.75)	576.08 (30.41)	491.53 (41.59)	<0.001	<0.001	<0.001
RT 86 ms	467.46 (34.13)	523.18 (30.76)	465.89 (42.07)	0.829	<0.001	<0.001
RT 114 ms	437.03 (32.93)	500.65 (29.68)	415.04 (40.59)	0.002	<0.001	<0.001
*Error rates*						
ER 14 ms	29.72 (4.13)	31.13 (3.72)	24.13 (5.09)	<0.001	<0.001	0.042
ER 29 ms	16.63 (3.81)	21.72 (3.43)	18.30 (4.70)	0.043	<0.001	<0.001
ER 43 ms	14.44 (3.18)	18.91 (2.86)	10.79 (3.91)	<0.001	<0.001	<0.001
ER 57 ms	9.10 (3.12)	13.82 (2.81)	11.78 (3.85)	<0.001	<0.001	<0.001
ER 86 ms	7.63 (2.84)	9.89 (2.56)	9.39 (3.50)	0.005	<0.001	<0.001
ER 114 ms	6.71 (2.94)	7.72 (2.65)	8.22 (3.63)	0.020	0.288	0.041

RT, reaction time; ER, error rate.

### Assessing the influence of duration of illness

In some items, we noted a significant but not substantial influence of DOI on cognitive performance. Correlation coefficients were found between 0.034 and 0.328 for the CVLT items, between 0.103 and 0.512 for the WAIS-R items and between 0.279 and 0.375 as well as 0.273 and 0.391 for the VBM variables (reaction time and error rate, respectively). Correlations regarding the CVLT and WAIS data were negative - the longer the DOI lasted, the fewer correct answers provided. Correlations with the VBM data were positive - the longer the DOI lasted, the slower the patient’s reaction, and the more errors that occurred.

## Discussion

The main findings of this study are that (treated) patients with a long-standing bipolar disorder (presenting a duration of illness of about 20 years and at least five illness episodes in their history) and controls did not differ significantly in neurocognitive performance in overall cognitive function and verbal learning, recall, and recognition, regardless of whether lithium was part of their long-term treatment. The patients, however, revealed impaired early visual information processing compared to healthy controls, with the lithium-treated patients performing worse than those without.

In contrast to the CVLT results from bipolar patients published by Martinez-Aran et al. (2004) and Fleck et al. (2003) (upon which we based our sample size estimation), our patients were a total of only about 6% worse than the healthy controls. Both patients and controls in the Martinez-Aran et al. study performed worse than ours, i.e., in the total number of words remembered on list A (45.1 ± 11.4 vs. 58.6 ± 14.0 and 54.4 ± 9.6 vs. 59.2 ± 17.1, respectively). Patients in the Martinez-Aran study were roughly comparable to ours with regard to age (about 8 years younger), education, and duration of the disease (about 5 years shorter). Their controls were also roughly comparable to ours regarding age and education. In terms of medication status at testing, 83% of their patients were taking lithium (vs. 65% in our total patient sample), 30% carbamazepine (vs. 11%), and 18% valproate (vs. 15%). Twenty-eight percent of their patients received more than one mood-stabilizing drug (vs. 14%), 58% were treated with antipsychotics (38% with atypical ones vs. 22%), and 20% received antidepressants. Their patients’ medication status differs from our study’s both in the percentage use of drugs we allowed and in the use of medications on our exclusion list (20% received typical antipsychotics, and it is not known whether clozapine or tricyclic antidepressants were used). In the study by Fleck et al., their 14 euthymic bipolar patients were measurably worse in the total number of words remembered on list A of the CVLT than the patients in the Martinez-Aran study (46.4 ± 14.3), whereas their controls were nearly as good as ours (58.9 ± 8.1). The Fleck study patients were about 15 years younger than ours and thus had a shorter duration of illness, but they are otherwise similar to ours with regard to education. Of Fleck’s cohort, 79% were treated with mood stabilizers and 50% with antipsychotics; however, they do not provide greater detail on the type of medication administered, which hinders comparison with our patients. In the meta-analysis by Robinson et al. (2006) (including the aforementioned studies), euthymic bipolar patients did display moderate to severe impairment in verbal learning and memory (and executive functioning) compared to healthy controls (effect sizes of 0.7 to 0.9). Compared to patients with schizophrenia, bipolar patients (including those in our study) seem to be less severely impaired in cognitive performance, and they reveal a similar pattern of functions affected (see Daban et al. 2006 and Trivedi et al. 2007). In a recent large individual patient data meta-analysis including a part of our sample, Bourne et al. showed only moderate effect sizes of neurocognitive impairment (for the CVLT 0.51, Bourne et al. 2013). They suggested that better control of the influence of age, gender, and IQ as well as the inclusion of unpublished data could explain the lower effect sizes at least in part.

Our results are in line with published VBM results in that euthymic bipolar patients were significantly slower and made more errors than controls (see for instance Green et al. 1994a, Green et al. 1994b, MacQueen et al. 2001). Contrary to our hypothesis, however, our lithium-treated patients demonstrated worse early information processing. This, however, seems to support the data from Fleming and Green, who observed impairment in backward masking to be partly associated with lithium treatment (in that bipolar patients on lithium present a significantly higher critical inter-stimulus interval than patients not on lithium, the latter still performing non-significantly worse than healthy controls (Fleming and Green 1995)).

We wish to stress our initial hypothesis in several ways: (A) Was it reasonable to suggest that phenomena such as the neuroprotective effects identified in preclinical studies and decreased dementia rates found in lithium-treated patients are associated phenomena and that these can be examined by applying neurocognitive performance tests, in this age group, and in conjunction with the duration of illness our patients presented? (B) Might lithium’s potential neuroprotective benefit compensate for the cognitive side effects seen with lithium treatment? We could also query (C) whether our patient control group (non-Li) might have differed from the lithium group and whether they might have received treatments more frequently that might themselves exert a neuroprotective effect.

Regarding hypothesis A and based on the results we present: we suggest that effective phase prophylaxis can reduce impairment in cognitive performance (independent of whether patients respond to lithium or to another mood-stabilizing medication). Rybakowski and Suwalska (2010) reported good cognitive functioning in conjunction with long-term lithium treatment in excellent responders. Lithium’s additional specific neuroprotective effects might compensate for cognitive functioning impairment due to the illness’s neurodegenerative or toxic effects until brain areas are on the verge of substantial damage (and dementia). Drawing on the data from their Danish registry, Kessing et al. (2010) demonstrated a reduced risk for dementia only in those patients on lithium treatment, but not when taking the other drugs studied (i.e., anticonvulsants, antipsychotics, antidepressants). Volumetric results from our study show, in line with our hypothesis, that the lithium-treated patients’ hippocampal volumes were larger than those of non-Li patients (who received the above-mentioned alternative mood stabilizing medication) and were similar to those of healthy controls, independent of long-term treatment response including the number of episodes while on lithium (Hajek et al. 2013). Moreover, our non-Li group patients’ prefrontal NAA levels were lower, while the Li group’s were similar to those in the healthy controls (Hajek et al. 2012), with NAA assumed to be a marker of cellular integrity. Since general cognitive functioning and verbal learning and memory were not substantially impaired in our non-Li patients, the smaller hippocampal volumes might still suffice for functioning, not causing a substantial impact until a greater loss of hippocampal volume has occurred.

Regarding hypothesis B: We suggest that we still observed minor cognitive impairment in our long-term ill lithium-treated bipolar patients compared to healthy controls because lithium side effects were balanced but not outweighed by potential neuroprotective effects. In their meta-analysis, Wingo et al. showed that lithium treatment was associated with rather minor cognitive impairment (Wingo et al. 2009). In the two longitudinal studies included in this meta-analysis, the cognitive performance over time was somewhat stable in the lithium-treated patients (Smigan and Perris 1983, Engelsmann et al. 1988), in line with our study’s result of an only insubstantial influence of duration of illness on overall cognitive performance, verbal learning, and memory as well as early visual information processing (though keeping in mind that we had little variance in duration of illness because of our inclusion criteria). Results from a recently published prospective study showed that neurocognitive performance of bipolar patients on lithium (monotherapy in half, combination therapy with AD or neuroleptics in the other half) was stable over the 6 years of follow-up (Mora et al. 2013). However, as in the aforementioned studies by Martinez-Aran et al. and Fleck et al., their patients were more severely impaired than ours (CVLT total words remembered of list A: baseline 51.5 ± 10.0, follow-up 6 years 49.4 ± 12.1).

To question hypothesis C: Every clinician agrees that patients that do not receive or sufficiently respond to prophylactic lithium treatment differ in some characteristics from patients that tolerate lithium and respond adequately. Contraindications to lithium treatment were of course more prominent in the non-Li group; however, we did not detect major differences in the patient groups’ comorbidity profiles. We matched them for potential influencing characteristics and required the same duration of illness and minimum number of previous episodes. Regarding medication however, the groups differed substantially with respect to the use of, for example, valproate (35% in the non-Li vs. 5% in the Li group), which may exert a potential neuroprotective effect itself (via modulating for instance the WNT pathway, also a major target of lithium, Sutton and Rushlow 2011). However, the clinical evidence does not support this suggested effect showing no increase in gray matter volume (Lyoo et al. 2010) or even greater loss in hippocampal and whole-brain volume (Tariot et al. 2011) with valproate. It is thus not clear whether the similar overall cognitive performance and that in verbal learning and memory could in part result from the use of protective medication in the patient control group.

### Strength and limitations

The present study applied strict inclusion and exclusion criteria. We only included patients in the Li group who had been taking lithium for at least the previous 2 years. In the non-Li group, a maximum of less than 3 months lifetime use of lithium was allowed, and treatment had to have been discontinued more than 2 years prior to the study’s beginning. The reason for these criteria was that we are unsure how long the potential neuroprotective effect (of lithium) takes to unfold. With the medication exclusion list we applied, acute impairing influences of drugs on cognitive performance were prevented. By allowing for only a limited number of drugs as comedication, we aimed to be able to ascribe effects to lithium in the Li group and to circumvent problems with multiple drug treatment. The sample sizes of our patient groups and controls are higher than those in most comparable studies.

Limitations include (a) the design of a cross-sectional study (here with inclusion of retrospective information) which is valid for specifying the hypothesis but bears a high potential for bias and does not allow causation to be substantiated and (b) the recruitment of long-term ill patients that did not have a substantial history of lithium treatment was a major task in the study. In the end, relatively few patients could be included in this group. This can be partially explained by the IGSLi centers involved in the study where expert clinicians are working in the treatment of BD and who are engaged in clinical and scientific research on the effects of lithium. (c) The study was conducted in five centers in different countries, where treatment and care settings as well as patient characteristics might well have varied. Our analysis was therefore adjusted to control for the influence of center effects. (d) As mentioned above, in the patient control group (non-Li), potentially neuroprotective drugs were used more frequently than in the lithium group, i.e., valproate, a circumstance however, that cannot be easily avoided in a future study since the patients who were included had been ill for a long time and needed prophylactic treatment. Additionally (e), when designing and conducting the presented study, we suggested a somewhat homogeneous distribution of cognitive impairment in the group of patients with bipolar disorder as a whole. However, results of a recently published cross-sectional study show that although patients with bipolar disorder as a group showed more cognitive impairment compared to healthy controls, only a third of the patients showed more severe impairment, whereas 70% showed either impairment of smaller effect or was indistinguishable from controls (Martino et al. 2014).

## Conclusions

Our data suggest that bipolar patients with a long illness history and effective prophylactic treatment do not show significantly impaired general cognitive functioning or verbal learning and memory, but that they do display worse early information processing. In the latter, the lithium-treated patients performed worst. Accompanying volumetric and spectroscopic data suggest cell loss in patients not treated with lithium, potentially counterbalanced by long-term lithium treatment.

At the moment, a follow-up assessment of the patients and controls presented here is being carried out at the respective IGSLi centers to prospectively investigate the effects of long-term lithium treatment in this very specific cohort of patients.
